# Smartphone Location Recognition: A Deep Learning-Based Approach

**DOI:** 10.3390/s20010214

**Published:** 2019-12-30

**Authors:** Itzik Klein

**Affiliations:** Huawei, Tel-Aviv Research Center and Department of Marine Technologies, University of Haifa, Haifa 3498838, Israel; Itzik.klein@huawei.com

**Keywords:** pedestrian dead reckoning, deep learning, accelerometers, human activity recognition

## Abstract

One of the approaches for indoor positioning using smartphones is pedestrian dead reckoning. There, the user step length is estimated using empirical or biomechanical formulas. Such calculation was shown to be very sensitive to the smartphone location on the user. In addition, knowledge of the smartphone location can also help for direct step-length estimation and heading determination. In a wider point of view, smartphone location recognition is part of human activity recognition employed in many fields and applications, such as health monitoring. In this paper, we propose to use deep learning approaches to classify the smartphone location on the user, while walking, and require robustness in terms of the ability to cope with recordings that differ (in sampling rate, user dynamics, sensor type, and more) from those available in the train dataset. The contributions of the paper are: (1) Definition of the smartphone location recognition framework using accelerometers, gyroscopes, and deep learning; (2) examine the proposed approach on 107 people and 31 h of recorded data obtained from eight different datasets; and (3) enhanced algorithms for using only accelerometers for the classification process. The experimental results show that the smartphone location can be classified with high accuracy using only the smartphone’s accelerometers.

## 1. Introduction

An inertial navigation system (INS) contains three orthogonal accelerometers and gyroscopes (inertial sensors). The accelerometers measure the specific force vector while the gyroscopes measure the angular velocity vector [1]. Using the sensors’ measurements and initial conditions, the navigation solution (position, velocity, and attitude) can be calculated. In general, such a process requires several integrations on the measured vectors. The inertial sensors’ measurements contain noise and other error terms, thus when integrated, a drift in the navigation solution occurs. To circumvent this drift, INS is commonly fused with other sensors or data, such as global navigation satellite systems (GNSSs) [2].

However, GNSSs cannot be used in indoor environments since the radio signals from the satellite are blocked. A possible solution for navigating indoors was suggested using a shoe-mounted INS [3]. There, the inertial sensors are mounted on a shoe and zero velocity updates are applied to reduce the navigation solution drift [4]. Yet, this kind of solution cannot be applied using the smartphone inertial sensors, since the smartphone motion is not constrained. To enable smartphone-based navigation, pedestrian dead reckoning (PDR), a two-dimensional navigation framework, was derived [5]. Loosely speaking, a PDR algorithm has four parts: (1) User step detection, (2) step length estimation using accelerometers measurements, (3) heading determination using gyroscopes measurements, and (4) given initial conditions, position and heading update. Compared to the INS algorithm, PDR requires less integrations on the inertial measurements. To that end, one of the major aspects of PDR is the estimation of the gravity direction for accurate heading determination and step length estimation [6]. Another dominating aspect is the step-length estimation. Usually, such estimation is based on empirical or biomechanical models [7], which requires an a priori calibration process to define the step-length estimation parameters (or gains). These estimation parameters and gains are very sensitive to pedestrian and smartphone modes [8]. Erroneous gain will yield an error in step length estimation, which in turn will produce a position error. That is, for accurate PDR positioning, different gain values are required for every pedestrian mode (such as walking, running, standing, elevator, etc.) and smartphone location (hand-held in a pocket, etc.).

Most of the literature on the subject address user mode recognition since it is a subset of a wider research topic known as human activity recognition (HAR) [9]. HAR is used in many fields and applications, such as health monitoring [10], smart homes [11], sports [12], and much more. For PDR applications, HAR approaches are used to identify the user mode, such as walking, running, standing, and others. For example, Qain et al. [13] used statistical measures to distinguish between walking and running modes followed by a selection of appropriate model parameters. In [14], four types of user modes—walking, running, bicycle, and vehicle—were classified using classical machine learning approaches. Recently, two comprehensive survey papers on user mode recognition [15,16] summarized published results on various motion mode types, including (but not limited to) walking, running, and stairs modes, which are most relevant for PDR applications.

Similar to HAR, smartphone location recognition (SLR) refers to the process of identifying the location of a smartphone on the user. The location of the smartphone changes due to the user actions. For example, consider a user holding his smartphone in his hand while walking—swing mode. Then, the user receives a phone call, thus the smartphone is moved near the user’s ear to talking mode. After completing the phone call, the user can put the smartphone in front and write a text massage—texting mode—or put the smartphone in his pocket—pocket mode. Those are the four main possible locations of a smartphone on a user. One of the earliest papers in the subject was SmartMTra [17] that addressed eight types of pedestrian modes and seven types of smartphone modes, where a distinction was made for texting with one or two hands and a big or small arm swing. Later, Susi et al. [18] proposed to distinguish between four cases: Static user, quasi-stable device to include texting, talking and bag carrying, and swing and irregular motion. In [19], a finite state machine was used to classify three smartphone modes: Swing, holding (texting), and pocket. Yand and Wang [20] designed a random forest classier to classify four smartphone modes, hand (swing), bag, coat pocket, and trouser pocket, to achieve an accuracy of 93.7%. To assist the integration of the accelerometer and gyroscope pedometers, a threshold-based approach was applied to distinguish between the presence and absence of a swing mode [21]. Klein et al. [22] used only the magnitudes of the gyroscopes’ and accelerometers’ measurements to classify the smartphone modes. Recently, in [8], the importance of gain selection for each smartphone mode was demonstrated in terms of PDR positioning errors. Using tree-based approaches, an accuracy of 95.4% was obtained for smartphone mode recognition. In the same manner, [23] proposed the use of neural network approaches for SLR but without defining a proper framework and on a limited dataset. Smartphone mode recognition was also applied in [24] to assist in a machine learning regression approach for step-length estimation. Also, in [25], smartphone mode recognition was used as a part of an algorithm to enable accurate heading determination.

In this paper, we define the SLR framework and propose deep learning approaches to perform the SLR task. We focus on a scenario when the user is walking with four possible smartphone locations: Talking, pocket, swing, and texting.

The contributions of the paper are:

1. Definition of the SLR framework using deep learning. In general, the smartphone accelerometers’ and gyroscopes’ measurements are plugged into a deep learning architecture, which in turn outputs the SLR result. To that end, four different deep learning-based architectures are considered.
2. Examination of the proposed SLR approach on 107 people and 31 h of recorded data. The number of different people who participate in this is dataset is 6 times more than any other dataset used in SLR (17 people in [19]) and the recording time is 12 times more than any other dataset used in SLR (about 160 min in [22]). This dataset was partly generated for this research and partly uses other publicly available datasets created for other applications but are suitable for the SLR task.
3. Robust classifiers for SLR. Usually, a single dataset is used for training and testing the classifier. Such a classifier is not robust to other datasets, which were not included in the training phase. The solution is to combine several datasets (here, three are used) in the training set in order to create a robust classifier to cope with unseen datasets. Such classifiers are provided here when using accelerometers’ and gyroscopes’ data as inputs and also when using only accelerometers for the SLR tasks.
4. A feasibility study to handle unknown smartphone locations. A binary network is proposed to determine using accelerometer data only if the current smartphone location is one of the four possibilities (texting, talking, swing, and pocket) or an unknown location. If the result is true (location is one of the four possibilities), the accelerometer data is passed to the proposed SLR architecture to determine the current smartphone location. Otherwise, the output of the binary network will be false, meaning the smartphone location is labeled as unknown.

The rest of the paper is organized as follows: Section 2 presents the SLR framework, including the datasets and deep learning architectures. Section 3 presents the results obtained using a single user for training while Section 4 does the same for multiple users. Finally, Section 5 gives the conclusions of this study.

## 2. Smartphone Location Recognition Framework

### 2.1. Framework

**Figure 1:**
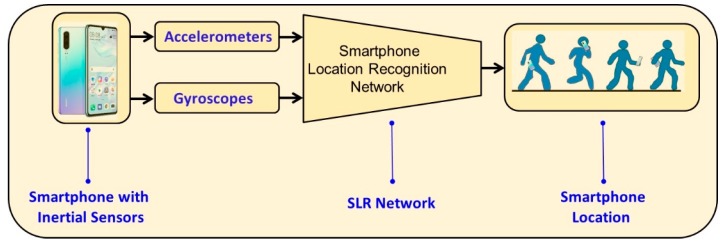
SLR framework block diagram. The input to the network is the inertial sensors’ measurements and the output of the network is the smartphone location.

A block diagram of the proposed SLR approach is presented in Figure 1. The smartphone inertial sensors, namely the accelerometers and gyroscopes, measure the specific force and angular velocity vectors. Those measurements are used as input to the SLR network. The output of the network is the smartphone location on the user. Four commonly used smartphone locations are considered: (1) Pocket: The smartphone is in the trouser pocket and free to move. The pocket can be in the front or back, left or right; (2) talking: The smartphone is placed in the user’s hand near the user’s ear. Talking can be performed close or far from the user’s ear and can be held in the right or left hand; (3) texting: The smartphone is handheld in front of the user approximately at waist height. The smartphone can be in a horizontal position for texting or almost vertical for other applications. Also, the smartphone can be held in the right or left hand and even with two hands; and (4) swing: The smartphone is handheld while the user is walking. The smartphone can be held in the right or left hand.

The proposed framework is applicable for any type of smartphone equipped with accelerometers and gyroscopes. We shall also show that accelerometers are good enough for the SLR procedure. In addition, there is no limitation on the recording application used to record the data.

The angular velocity measurements were not normalized since the measurements contain noise and other error terms, which can be mistreated by the classifier, if normalized, particularly in situations when the actual angular velocity can be near zero (or in the same order of the noises), such as in texting or talking. On the other hand, the same phenomena are not valid for the accelerometers’ measurements since the gravity is a bigger quantity (1 g) than the accelerometer noise and other error terms (mg) and also due to the acceleration the pedestrian experiences while walking. Therefore, we followed the common practice in machine learning to normalize the accelerometer recordings.

**Figure 2:**
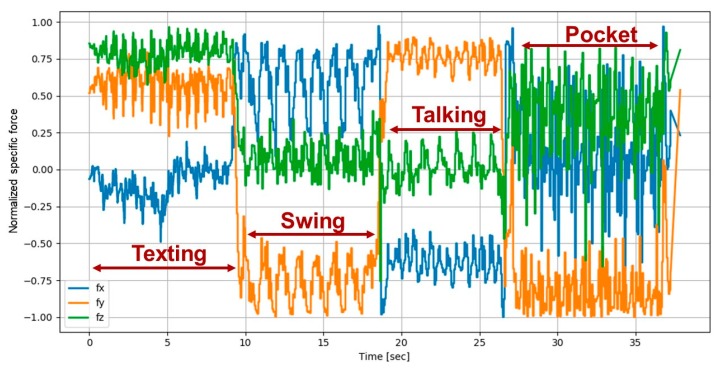
Normalized specific force vector as recorded during four smartphone locations: texting, swing, talking, and pocket.

An example of the specific force vector as measured by smartphone accelerometers is shown in Figure 2. The difference between the four smartphone modes in the recordings can be clearly seen in the figure. Notice, that the accelerometers raw data was normalized using the Euclidean norm. Given the measured specific force vector:

(1) $$f=\;{\left[\begin{array}{ccc} f_{x} & f_{y} & f_{z} \end{array}\right]}^{T}.$$

The normalized specific force vector is:

(2) fn=[fx|f|fy|f|fz|f|]T,

where:

(3) $$\left|f\right|=\sqrt{f_{x}^{2}+f_{y}^{2}+f_{z}^{2}}.$$

The normalized specific force, Equation (2), is used as input to the SLR network.

**Figure 3:**
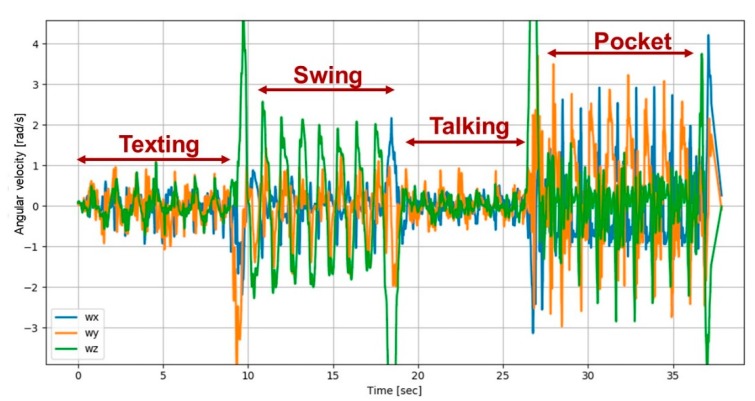
Angular velocity vector as recorded during four smartphone locations: texting, swing, talking, and pocket.

In the same manner, an example of the angular velocity vector as measured by smartphone gyroscopes is shown in Figure 3. The difference between the four smartphone modes in the recordings can be clearly seen in the figure. As expected, during the texting and talking modes, the angular velocity oscillates around zero. Thus, if normalized, it would blur the difference between the four smartphone locations and as a consequence harden the classification process. Therefore, the measured angular velocity:

(4) $$\omega ={\left[\begin{array}{ccc} \omega_{x} & \omega_{y} & \omega_{z} \end{array}\right]}^{T},$$

is used as input to the SLR network.

Key points of the proposed framework include the following:

(1) The input to the SLR network is the normalized specific force vector (Equation (2)) and the angular velocity vector (Equation (4)).
(2) The output of the SLR network is the smartphone location on the user, which is one out of four possibilities: Talking, texting, swing, and pocket.
(3) Besides the specific force normalization, no other operations are made on the raw data.
(4) No measurement rejection algorithm or noise reduction approaches were applied.
(5) The proposed framework was examined for a scenario in which the user is walking. Walking speed varied between slow to normal to fast.
(6) The proposed approach is valid for any smartphone type and for any recording application.
(7) The dataset recordings were obtained in different sampling rates, from 20 to 200 Hz, to make the proposed deep learning architecture robust to the sampling rate.

### 2.2. Deep Learning Architectures

We consider four types of neural networks architectures: (1) Long- and short-term memory (LSTM) recurrent neural network; (2) one-dimensional convolutional neural network (CNN); (3) gated recurrent unit (GRU) recurrent neural network; and (4) CNN/LSTM network. Similar network architectures were shown to perform well in human activity recognition (e.g., [26]). Since SLR is a similar task to HAR, we assumed similar network architectures will also be able to perform well in SLR.

**Figure 4:**
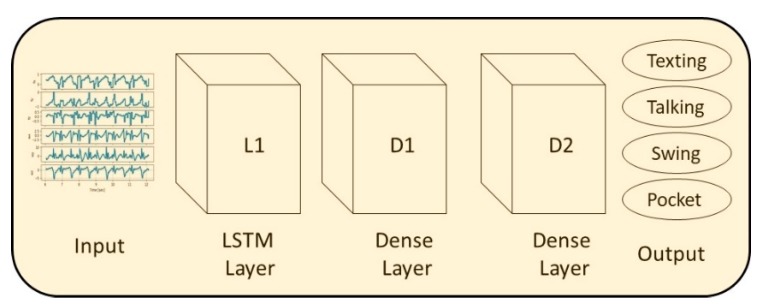
The LSTM network architecture used for evaluation of SLR.

In general, LSTM is a variation of recurrent neural networks, which solves their vanishing gradient problem of learning long-term dependencies by using a gating mechanism [27]. The LSTM architecture that was used for the evaluation of SLR is presented in Figure 4. It receives the specific force and angular velocity vectors as input to the first LSTM layer (L1). The second layer is a fully connected layer (D1) as well as the third layer (D2), which outputs the smartphone location. The default parameters for the network were set as follows: L1 has 128 units, D1 has 32 with a rectified linear unit (ReLU) activation, and D2 has 4 with Softmax activation.

**Figure 5:**
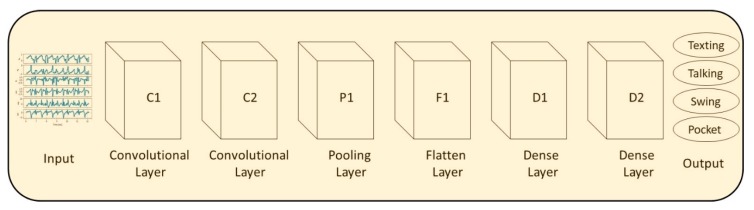
The CNN network architecture used for the evaluation of SLR.

One-dimensional CNN are simply neural networks that use convolution in place of general matrix multiplication in at least one of their layers. The convolutions are used as layers to filter inputs for useful information/features for the required task [28]. The CNN architecture that was used for the evaluation of SLR is presented in Figure 5. It receives the specific force and angular velocity vectors as input to the first CNN layer, C1. C1 has 32 units with an ReLU activation. The next layer is also a 1D-CNN, C2, with the same parameters as C1. After dropout of 0.6, the next layer is a polling layer of size 2 followed by a flattened layer to set the dimensions to the following dense layer, D1. D1 has 32 units with an ReLU activation function followed by a second dense layer, D2, with Softmax activation that outputs the SLR result.

**Figure 6:**
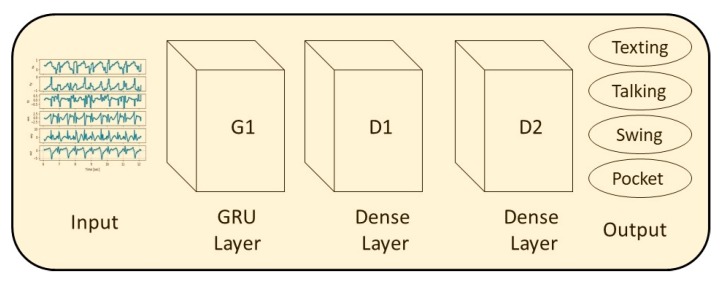
The GRU network architecture used for the evaluation of SLR.

GRU is basically an LSTM without an output gate. The main difference with the LSTM is that a single gating unit simultaneously controls the forgetting factor and the decision to update the state unit [27]. The GRU architecture that was used for the evaluation of SLR is presented in Figure 6. It receives the specific force and angular velocity vectors as input to the first GRU layer. Then, just like in the LSTM architecture, the second layer is a fully connected layer as well as the third layer, which outputs the smartphone location. The default parameters for the network were set as follows: G1 has 128 units, D1 has 32 with ReLU activation, and D2 has 4 with Softmax activation.

A CNN/LSTM architecture combines both CNN (as the first layer) followed by an LSTM layer. CNN has the ability to extract features from the data while LSTM can explore temporal dependencies in the time series problem. Thus, when combined, one can enjoy both of their benefits. Such a kind of architecture is applied for various applications, including HAR [26].

**Figure 7:**
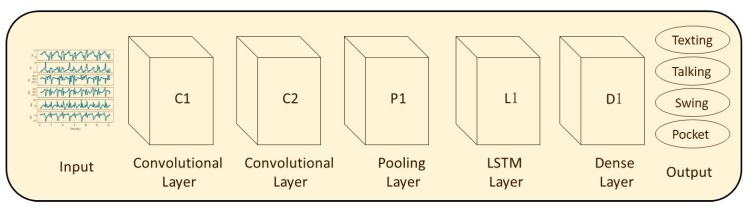
The CNN/LSTM network architecture used for the evaluation of SLR.

The CNN/LSTM architecture that was used for the evaluation of SLR is presented in Figure 7. It resembles the CNN architecture (Figure 5) except that the flattened layer and the first dense layer are replaced with an LSTM layer with 32 units.

All four network architectures were implemented using Keras open-source neural network library in Python [29] with a Windows environment. In all networks, the loss function was categorical cross entropy [30] (CCE) for a single label categorization. The optimization was performed with the RMS propagation [31] algorithm (RMSProp), which divides the gradient by a running average of its recent magnitude. The main network parameters are summarized in Table 1.

### 2.3. Datasets

To evaluate the proposed SLR framework, eight different datasets were used. Two of the datasets were constructed directly for this research while the other six datasets that were found in the internet were constructed for other applications, like HAR or PDR. In all the datasets, the smartphone location was in one of the four categories (pocket, text, swing, and talk) while the users were walking. Additionally, there was no limitation on how the smartphone should be used in each location. For example, texting can be made while the user is holding the phone in the left hand and texting with the right or even holding the phone in two hands.

The first dataset, constructed for this research, contains only a single user’s recordings. The recordings were made in a range of sampling rates between 25 and 100 Hz, with a total recording time of about 164 min. This dataset, denoted as U1, contains all four possible smartphone locations that were recorded in inhomogeneous conditions. Those include varying walking speeds, walking on uneven pavements, transitions between pavement and roads, varying hand swing (small to big), tight and sport trousers with a front and back pocket location, and texting and talking with a single hand (right and left) in different positions relative to the user.

**Figure 8:**
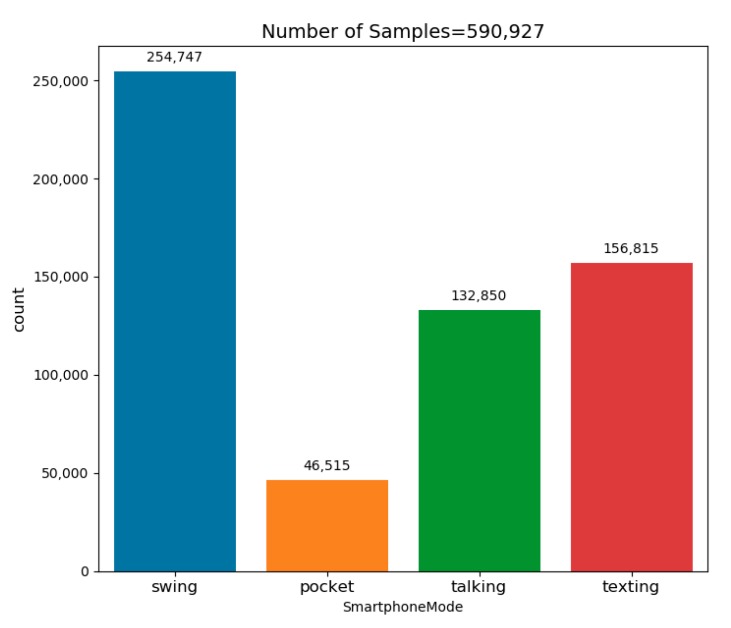
Dataset U1 sample’s distribution to each smartphone location.

The number of samples in each possible smartphone location are presented in Figure 8. Additionally, the U1 dataset’s main parameters as well as all other dataset parameters are summarized in Table 2.

**Figure A1:**
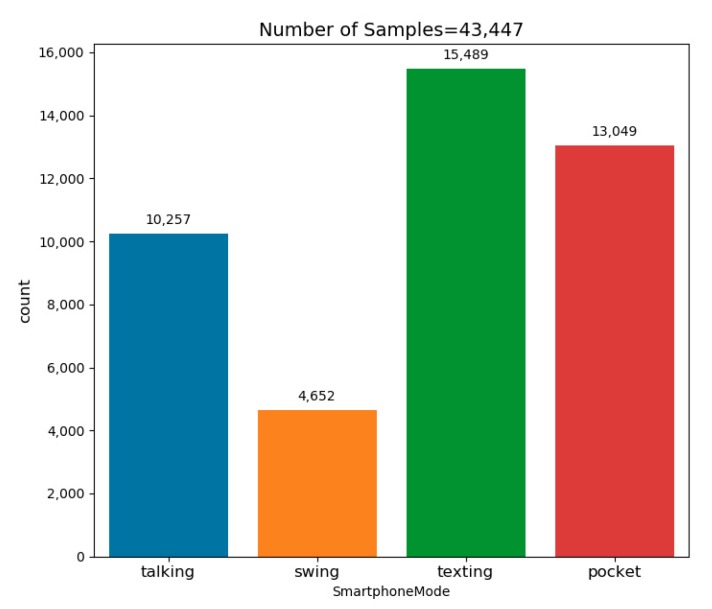
The HTA dataset’s sample distribution to each smartphone location.

The second dataset was constructed with the help of the Huawei’s Tel-Aviv research center people. The number of participants is six, five men and one woman. Each participant used a different smartphone for the recordings. The sampling rate was between 25 and 100 Hz, and contained all four smartphone modes, with a total time of about 15 min. The dataset’s, denoted as HTA, main parameters are given in Table 2 and its sample distribution is presented in Appendix A, Figure A1.

**Figure A2:**
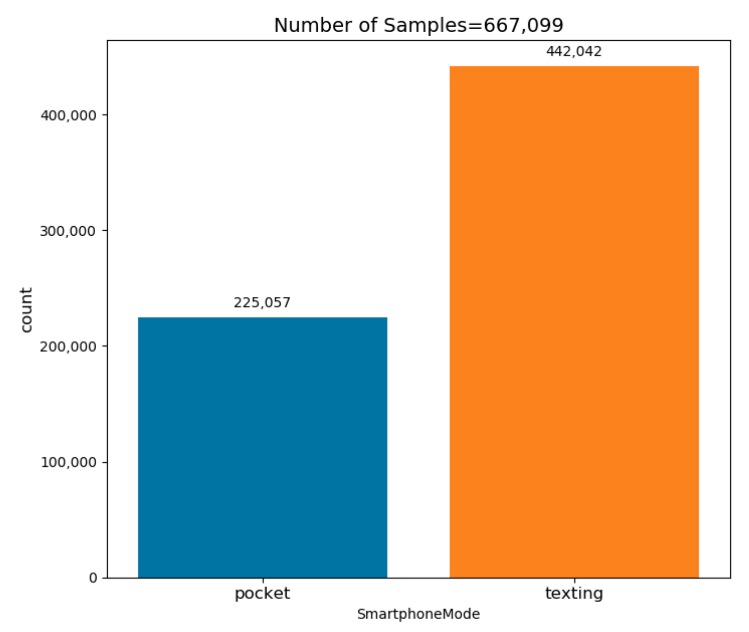
The RIDI dataset’s sample distribution to each smartphone location.

The third dataset was provided by [32]. The objective there was to drive a machine learning approach to better estimate the acceleration change, to correct the raw accelerometer data and then perform double integration to obtain the position. Their work is not related to SLR, yet their dataset was recorded using a smartphone in the front pocket and also in texting mode with eight participants. We employed the dataset for our SLR evaluation. This dataset is denoted as RIDI. Its main parameters are given in Table 2 and its sample distribution to each of the smartphone locations is presented in Appendix A, Figure A2.

**Figure A3:**
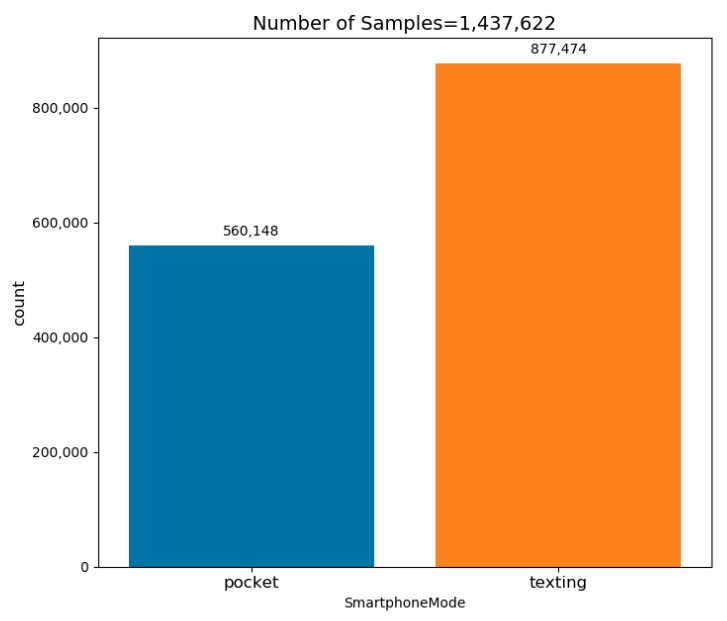
The OXF dataset’s sample distribution to each smartphone location.

In the same manner, [33] proposed the use of deep learning to calculate the pedestrian position and heading instead of classical PDR algorithms. To that end, they constructed a dataset using seven participants that recorded data for 240 min with the smartphone in the pocket or texting mode. This dataset is denoted as OXF and its main parameters are also given in Table 2. The sample distribution of the OXF dataset is presented in Appendix A, Figure A3.

The fifth dataset [34] was used to examine a new approach to a multi-objective loss function for training deep autoencoders for HAR applications. The dataset was recorded by 24 people (10 women and 14 men) using a smartphone in their pocket. Several activities were recorded, and only walking was employed herein. This dataset is denoted as MSR and its main parameters are given in Table 2. In the MSR dataset, all samples were recorded when the smartphone was located in the front pocket of users wearing tight trousers.

The sixth dataset was also employed from an HAR paper [35]. Their goal was to recognize user motions, such as walking, jogging, stairs, and standing, using only accelerometers. Herein, only the recordings of walking motion were considered. This dataset is denoted as WIS and its main parameters are given in Table 2. All the recordings of the accelerometers’ data were made when the smartphone was located in the front pant’s leg pocket.

**Figure A4:**
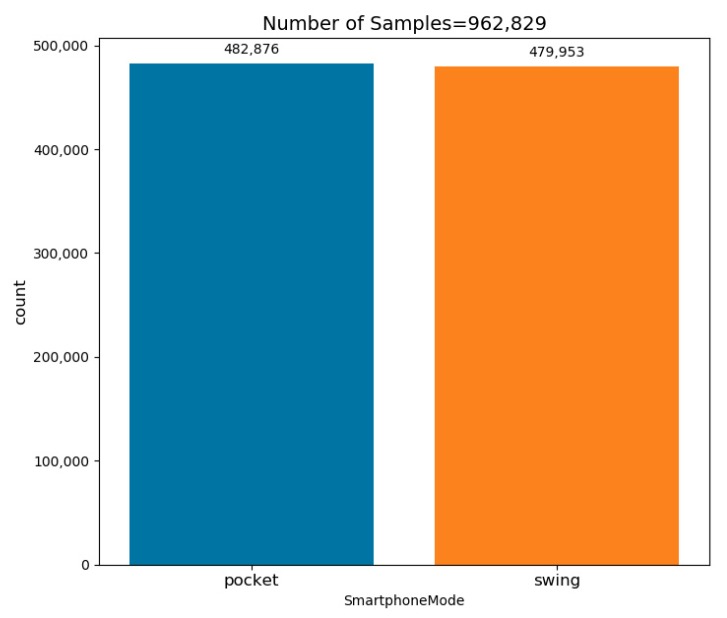
The WOB dataset’s sample distribution to each smartphone location.

The seventh dataset was also employed from an HAR paper [36]. There, the authors used wearable sensors located in seven positions on the user: Chest, forearm, head, shin, thigh, upper arm, and waist for HAR. We used the recordings during walking from smartwatches since they have the same dynamics as a smartphone in a swing motion and also from the sensors located at the thigh as they have the same dynamics as in the smartphone pocket mode. The dataset was recorded by 15 people (7 females and 8 men). This dataset is denoted as WOB, and its main parameters are given in Table 2 and its sampling distribution is presented in Appendix A, Figure A4.

The eighth dataset was employed from an HAR research [37]. There, the goal was to show HAR (walking, stairs, bike, and more) using smartphones’ and smartwatches’ data. The recordings during walking from the smartwatches were employed since they share the same dynamics as smartphones in a swing motion. In their research, the smartphone was located in a jeans pocket but was forced to be fixed, resulting in an unnatural behavior for the pocket mode. Therefore, the pocket dataset was not used in this work, leaving only the swing location samples. This dataset is denoted as PAR and its main parameters are given in Table 2.

**Figure 9:**
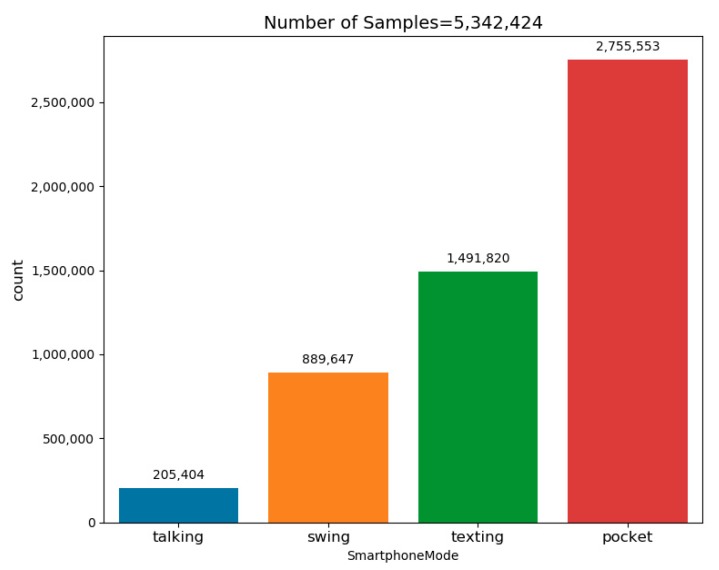
The final dataset’s (comprising of the eight different datasets) sample distribution to each of the possible smartphone locations.

To summarize, eight different datasets were used to evaluate the proposed approach. This combination of datasets resulted in the largest dataset that has been used for the purpose of SLR. It contains 107 people with about 31 h of recording, with varying recoding conditions and user walking dynamics. The distribution of the samples from all datasets to the four smartphone locations is presented in Figure 9. Since the dataset was constructed from smaller different datasets, not necessarily constructed for the SLR task, the distribution is not balanced between the four modes. Nevertheless, each smartphone mode has more than 200,000 samples.

**Figure 10:**
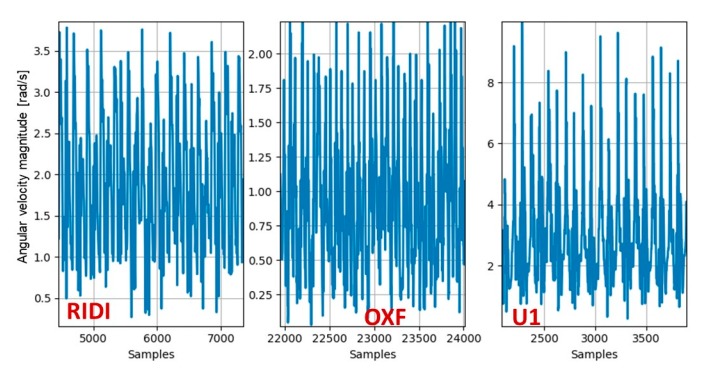
The angular velocity magnitude for a smartphone in the pocket mode as recorded for three users, each from a different dataset—RIDI, OXF, and U1.

Each user has different dynamics even in the same smartphone location. An example is presented in Figure 10. It was produced for a smartphone in the pocket mode from three users, each one from a different dataset (RIDI, OXF, and U1). The plots show the angular velocity magnitude for a random portion of samples. As can be seen, the maximum value and other statistical properties differ between the three.

### 2.4. Training Approach

Two types of training modes were considered. The first, following [8], is a single user training mode. The motivation is clear, as it has practical considerations. It is easier to collect data from a single user then from multiple users. To that end, the U1 dataset was divided into two parts for the training and testing labeled as TrainU1 and TestU1, respectively. The number of samples used in each part is presented in Table 3 for each smartphone location. There, the percentage is the ratio (test and train parts) from the original dataset, and in the parentheses are the number of samples. The second mode is multiple-user training. The motivation steams from a deep-learning perspective stating that as the number of users increases, the network will be able to learn mode differences much more efficiently and as a consequence will be more robust to other users who did not participate in the training process. To that end, both RIDI and OXF datasets were divided to train datasets (TrainRIDI and TrainOXF) and test datasets (TestRIDI and TestOXF). The number of samples used in each dataset is also presented in Table 3.

Combining TrainRIDI and TrainOXF with TrainU1 gives the multiple-user training dataset denoted as TrainROU, which has 15 different recordings. This dataset has a total of about 204 min of training data. In the same manner, combining the TestRIDI, TestOXF, and TestU1 datasets gives the TestROU dataset, consisting of approximately 256 min of recordings. The total amount of time in minutes of each smartphone location in TrainROU and TestROU is presented in Table 3.

Notice that since the RIDI and OXF datasets contain recordings only in the pocket and texting modes, the training on swing and talking modes are based only on the U1 dataset.

In the process of constructing the TrainU1, TestU1, TrainROU, and TestROU datasets, there was a clear distinction between the train and test files in a way that each file used for training was not used again for testing. As an example, a dataset containing three files—rec1, rec2, and rec3—was considered. One possibility for dividing the files is rec1 and rec2 for the train dataset while rec3 is for the test dataset.

All other five datasets were used only as test data to examine the performance of each network architecture, as proposed in Section 2.3.

Also, we note that all the train and test results presented in Section 3 and Section 4 were produced with a time window of 32 samples. That is, since the sampling rate is between 20 and 200 Hz, the SLR results will require a minimum of 0.16 s (for the 200 Hz sampling rate) to a maximum of 1.6 s (for the 20 Hz sampling rate).

Finally, the possibility of using all eight datasets and dividing them into test/train sets in a manner that recordings from each dataset will be present both in the train and test sets was not considered in this paper. The reason for that is that we aimed to examine the robustness of the proposed deep learning network to different recordings’ characteristics that were not available in the training process.

## 3. Results and Discussion—Single User Training

The TrainU1 dataset was employed for training while all other seven datasets and TestU1 dataset were used for testing. We employed the accuracy as the performance measure. The accuracy was defined as the total number of correct predictions divided by the total number of predictions. Section 3.1 shows the SLR results when using both accelerometers’ and gyroscopes’ measurements for training and testing while Section 3.2 does the same when using accelerometers’ measurements only.

### 3.1. Accelerometers and Gyroscopes

The test accuracy results when training on the TrainU1 dataset are presented in Table 4. CNN is the best architecture for this scenario, with a more than 97% accuracy. CNN/LSTM also obtained an accuracy above 91%. When testing on the other six datasets, the accuracy was less than 70% and therefore, it is not presented in detail. The conclusion from these results is that when training on a single user, an accuracy above 90% can be achieved only when testing on the same user (TestU1) or other users using the same sampling rate and recording application.

In some datasets, the samples are not balanced between the smartphone locations, thus when considering only the complete dataset accuracy (as presented in Table 4), misleading conclusions may be reached. To that end, the recognition accuracy of each smartphone location is presented in Table 5 for the HTA and TestU1 datasets. For all possible smartphone locations, the accuracy was above 95.7%.

### 3.2. Accelerometers Only

The test accuracy results when training on the TrainU1 dataset while using only accelerometers are presented in Table 6. As in the accelerometer and gyroscope scenario in Section 3.1, CNN is the best architecture, with an accuracy above 92%. Compared to that scenario, the CNN architecture results were lowered by several percentages (4%–5%) but are still satisfactory and above 90%.

The accuracy of each smartphone location is presented in Table 7 for the HTA and TestU1 datasets. All four smartphone locations were correctly classified, with an accuracy above 91%. Compared to the accelerometer and gyroscope scenario in Section 3.1, the accuracy was reduced between 3% and 6% in all smartphone locations when using the same network parameters.

### 3.3. Summary

In this section, only a single user dataset, TrainU1, was used for the training phase. For practical considerations, it is easier to collect data from a single user then from multiple users. An accuracy above 90% was achieved only when testing on the same user, TestU1, or other users using the same sampling rate and recording application. However, when testing on the other six datasets with 100 different recordings (and with different characteristics from the training dataset), the accuracy was less than 70% for both the accelerometer and gyroscope (Section 3.1) and accelerometer only (Section 3.2) scenarios. Hyperparameter tuning was made on the network parameters to improve the classification performance; however, with no success. Thus, the single user classifier was found to not be robust enough to cope with unseen datasets of different people and recording characteristics.

## 4. Results and Discussion—Multiuser Training

The TrainROU dataset was constructed from recordings of the TrainRIDI, TrainOXF, and TrainU1 datasets as presented in Table 3. This dataset contained recordings from 15 people. All the other five datasets and the reaming files in the RIDI, OXF, and U1 datasets were used for testing. Section 4.1 shows the SLR results when using both accelerometer and gyroscope measurements for training and testing while Section 4.2 does the same when using accelerometer measurements only.

### 4.1. Accelerometers and Gyroscopes

The test accuracy results when training on the TrainROU dataset are presented in Table 8. All four network architectures obtained an accuracy better than 90%. Notice that the HTA and MSR datasets are not included in the training set and still, the SLR accuracy is high. Particularly, when using the TrainU1 on the MSR dataset the accuracy was less than 70%. Using TrainROU led to an improvement of more than 20% while in both training sets, the MSR dataset was not included. The rest of the dataset contains accelerometer data only and therefore is not addressed in this section.

In addition, as expected, the accuracy on the U1, RIDI, and OXF files that were not included in the training dataset was above 98%. This result strengthens the conclusion from Section 3.1. That is, testing on the same group as of the training, but with different recording files, is capable of obtaining very high accuracy results.

Focusing on the CNN architecture, the accuracy of classifying each smartphone location is presented in Table 9 for five different datasets. In all datasets examined and in all smartphone locations, the accuracy of recognizing the true smartphone location is above 94.8%.

### 4.2. Accelerometers Only

Table 10 presents the test accuracy results when training on the TrainROU dataset. The last column in the table contains all recordings from the RIDI, OXF, and U1 that were not included in the training. As shown in the previous section, the accuracy is above 95% as they are from the same group. In addition, testing on the HTA, OWB, and PAR datasets obtained an accuracy greater than 90%. However, the accuracy on the MSR and WIS datasets is less than 83%.

The results obtained so far were achieved using default network parameters as described in Section 2.2. To enhance the performance of the networks against unseen datasets, in the following section, we preformed hyper parameter tuning on the network parameters. Particularly, the goal was to improve the results of the MSR and WIS datasets since in the other datasets, the performance was more than satisfactory. For example, with the CNN architecture, the accuracy of recognizing the true smartphone location was above 94.4% for the HTA, OWB, PAR, and ROU test datasets. Since hyperparameter tuning will be conducted on the networks in the following section, the accuracy of each smartphone location is not presented here but will be addressed in the following section using the optimal parameters found in the parameter tuning process.

### 4.3. Hyperparameter Tuning

In this section, limited hyperparameter tuning around the initial network parameters (Section 2.2) was conducted only for the CNN and CNN/LSTM architectures using the TrainROU dataset. To that end, the following hyperparameters were examined: (1) The number of units in the CNN layer (16, 32, and 48), (2) the number of units in the dense layer (32, 64, and 128), and (3) the dropout value (0.4, 0.5., 0.6, and 0.7). The results of the hyperparameter test are provided in Table 11. The CNN architecture improved the MSR performance by 10% to 84.5% while the WIS dataset remains almost unchanged. This came with a cost of reduction between 1% and 3% in the other datasets. On the other hand, the CNN/LSTM with the new parameters managed to improve all datasets. In particular, the MSR dataset was improved by 25% to 96.6% and WIS by 20.5% to 92.9%.

Focusing on the CNN/LSTM architecture, the accuracy of classifying each smartphone location is presented in Table 12 for six different datasets. In all possible smartphone locations, the accuracy is above 91.6%.

### 4.4. Unknown Smartphone Locations

In this section, a feasibility study to cope with unknown smartphone locations is presented. The proposed SLR architecture was shown to handle datasets that were not in the training set, but can this architecture handle other smartphone locations, such as a belt, bag, or waist? Of course, since those locations were not used as outputs, the proposed SLR architecture will not be able to classify them, rather it will be forced to treat them as one of the four locations it was trained to classify. Therefore, we designed a binary classifier to determine, using only accelerometers data, whether the current smartphone location was one of the four possibilities (text, talk, swing, and pocket) or an unknown location (belt, bag, and waist). If the result is true (location is one of the four possibilities), the accelerometers data is passed to the proposed SLR architecture, as derived in Section 4.3, to determine the current smartphone location. Otherwise, the output of the binary network will be false, meaning the smartphone location is labeled as unknown.

**Figure 11:**
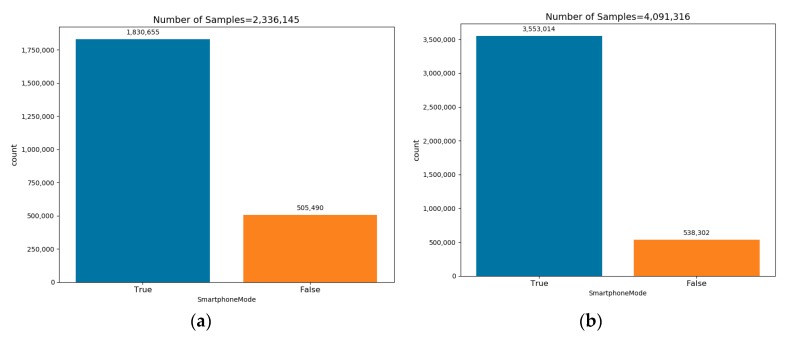
Train (**a**) and test (**b**) datasets’ sample distributions used for the binary classifier.

The training dataset for the binary network was the TrainROU dataset, now labeled as true with additional new recordings labeled as false, for the unknown smartphone locations. The latter consists of 10 recordings when the smartphone was on the waist taken from the OWB dataset, 7 recordings when the superphone was attached to the belt taken from the PAR dataset and from the OXF dataset, and 1 recording when the smartphone was placed in a bag. In the same manner, the testing dataset consists of the TestROU dataset, 6 recordings while the smartphone was on the waist from the OWB dataset, 3 recordings from the PAR dataset were the smartphone was attached to the belt, and 3 recordings from the OXF dataset when the smartphone was placed in the bag. The number of training and testing samples in the true/false modes are presented in Figure 11.

**Figure 12:**
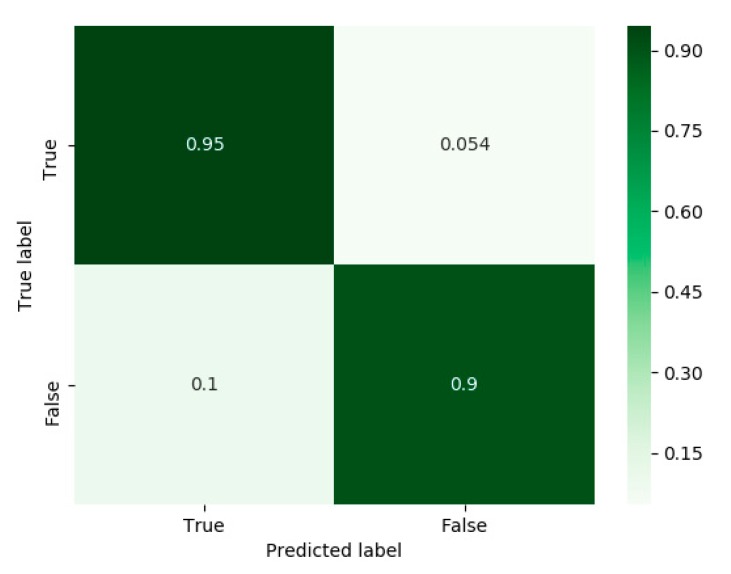
Confusion matrix plot for the binary classifier.

The binary network architecture was chosen to be a dense neural network with a structure of 128-64-1 units. The classification performance of the binary classifier is presented in Figure 12. As can be seen, it performs well in predicting both the true and false labels, with an accuracy of 95% and 90%, respectively. If a false label is predicted, the smartphone location is set to unknown; otherwise, when true label is detected, the proposed SLR architecture is applied to classify the smartphone location.

### 4.5. Summary

In this section, a dataset generated from three different datasets—RIDI, OXF, and U1—was constructed. The dataset contains recordings from 15 different people. When using accelerometer and gyroscope data, the accuracy in recognizing the smartphone location in six unseen datasets was above 94.8%. Those results were obtained without hyperparameter tuning. Repeating the process only with accelerometer data degraded the performance in some datasets to about a 70% accuracy. To circumvent that, hyperparameter tuning was conducted on the CNN and CNN/LSTM architectures. The results show an accuracy above 91.5% for all possible smartphone locations. Thus, a robust network architecture capable of coping with unseen datasets was found when using the accelerometer data only. In addition, a feasibility study presented an approach using a binary classifier before the SLR network to cope with unknown smartphone locations.

## 5. Conclusions

In the paper, the subject of smartphone location recognition was addressed and the SLR framework was defined. It states that when using inertial sensors (accelerometers and gyroscopes) and deep learning networks, the smartphone location on the user can be determined. Four different network architectures were used in the evaluation. The dataset was comprised of eight datasets, six of them are publicly available and two were constructed for this research. Among the six, four were constructed for HAR applications and two for deep learning-based PDR. In total, the dataset included recordings from 107 people, with a recording time of 31 h. In this dataset, four possible smartphone locations (talking, swing, pocket, and texting) were addressed while the users were walking.

The aim was to find a robust network capable of dealing with recordings that differ (in terms of sampling rate, user dynamics, sensor type, and more) from those available in the training dataset. Therefore, two training approaches were considered: (1) Single user from one dataset (U1) and (2) 15 users from three different datasets (ROU). This left five datasets not present in the training set for the robustness evaluation.

Using a single user in the training (TrainU1) enables a performance of more than 97% accuracy, but only on the same user additional test data (TestU1) or with other people in the group (HTA) using the same recording application and with similar recoding conditions. When testing on the other six different datasets, the accuracy was lowered to less than 70%, which means the network is not robust enough to handle recordings with different conditions, such as sampling rate or the type of recoding application. Next, 15 users’ (collected from three different datasets) recordings were used in the training (TrainROU). As in the single user case, results on the other test files of those users as well as on all other users in the datasets (TestROU) obtained an accuracy of more than 97%. This result, together with the one obtained for the single user training, leads to the immediate conclusion that it is possible to design a network to achieve excellent performance when using the same environmental and recording conditions both in the training and testing dataset. Yet, the main difference between the two training approaches was that when using the 15 training datasets (instead of a single user), the network was more robust to cope with the other five datasets that were not included in the training stage. In that case, an accuracy of more than 95% was achieved. Thus, we demonstrated that when using accelerometer and gyroscope measurements, the SLR problem can be solved even when training on a limited amount of people (14% of the total people).

This experiment was repeated but this time using only the smartphone accelerometers. From a machine learning perspective, since the amount of data is reduced by half (no gyroscopes) the problem at hand becomes more challenging. On the other hand, less data also means faster training and evaluation on real-time applications. Using the initial network parameters, a similar (to the accelerometer and gyroscope scenario) conclusion was drawn: It is possible to design a network to achieve excellent performance when using the same environmental and recoding conditions (same group) both in the training and testing dataset. When examining the other five datasets, an accuracy of more than 90% was achieved on three of them and on the other two a maximum accuracy of 83% was reached. To improve the latter result, limited hyperparameter tuning was applied on the network parameters. When doing so, the accuracy performance on those two datasets was improved to 93%. Thus, after some network parameter tuning, when examining the network of all five datasets that were not included in the training, an accuracy better than 92% was obtained.

To conclude, after deriving the SLR framework, we demonstrated that the problem of SLR can be solved for large-scale users, where the data was obtained from eight different datasets consisting of various recording conditions and with different environmental constraints. This was made possible using a robust network architecture that can be trained on a limited number of people using accelerometers and gyroscopes or accelerometer measurements only.

Additionally, a feasibility study of dealing with unknown smartphone locations was made. Therefore, an approach using a binary classifier before the SLR network to cope with unknown smartphone locations was suggested. For the unknown mode, recordings from the belt, waist, and bag locations were used. Results of the binary network showed a prediction accuracy of 95% and 90% for the true and false labels, respectively.

## Figures and Tables

**Table 1:** Main network parameters.

Architecture/Parameter	LSTM	CNN	GRU	CNN/LSTM
Batch size	32	32	32	32
Units	128-32-4	32-32-32-4	128-32-4	32-32-32-4
Activation	ReLU/Softmax	ReLU/Softmax	ReLU/Softmax	ReLU/Softmax
Loss function	CCE	CCE	CCE	CCE
Optimization	RMSProp	RMSProp	RMSProp	RMSProp

**Table 2:** Main parameters of all eight datasets used in the analysis. Smartphone locations are pocket (P), swing (S), texting (T), and talking (K). Sensors are accelerometers (Acc) and gyroscopes (Gyr).

Parameter/Dataset	U1	HTA	RIDI [32]	OXF [33]	MSR [34]	WIS [35]	WOB [36]	PAR [37]
Number of samples	590,927	43,447	667,099	1,437,622	329,960	1,097,948	962,829	88,000
Sampling rate [Hz]	25–100	25–100	200	100	50	20	50	50
Total time [Min]	~164	~15	~56	~240	~110	~915	~120	~29
Number of participates	1	6	8	8	24	36	15	10
Year	2019	2019	2018	2018	2018	2010	2016	2014
Smartphone Modes	P/S/T/K	P/S/T/K	P/T	P/T	P	P	P/S	S
Sensors	Acc/Gyr	Acc/Gyr	Acc/Gyr	Acc/Gyr	Acc/Gyr	Acc	Acc	Acc

**Table 3:** Samples and corresponding time distribution of each smartphone location in the two types of training modes’ datasets. The percentage is the ratio from the original dataset and the parentheses show the number of samples.

Smartphone Location/Database	Pocket	Swing	Texting	Talking
TrainU1	80% (37,380)	30% (68,927)	53% (83,152)	78% (102,706)
TestU1	20% (9135)	70% (185,820)	47% (73,663)	22% (30,144)
TrainRIDI	36% (80,067)	N/A	15% (63,816)	N/A
TestRIDI	64% (144,990)	N/A	85% (378,226)	N/A
TrainOXF	36% (201,247)	N/A	34% (296,642)	N/A
TestOXF	64% (358,901)	N/A	66% (580,832)	N/A
TrainROU	48.9 min	31.4 min	74.5 min	49.3 min
TestROU	74.9 min	31.1 min	139.5 min	10.6 min

**Table 4:** Test accuracy results when training on the TrainU1 dataset while using both accelerometer and gyroscope measurements.

Architecture/Database	HTA	TestU1
LSTM	82.3	93.8
CNN	97.5	97.1
GRU	77.4	94.7
CNN/LSTM	91.3	94.9

**Table 5:** Test accuracy results when training on the TrainU1 dataset with the CNN architecture while using both accelerometer and gyroscope measurements.

Database/Smartphone Location	HTA	TestU1
Pocket	98.8	98.3
Swing	97.2	96.5
Texting	95.7	97.9
Talking	96.9	98.2

**Table 6:** Test accuracy results when training on the TrainU1 dataset while using only accelerometer measurements.

Architecture/Database	HTA	TestU1
LSTM	90.0	92.3
CNN	92.9	92.3
GRU	79.4	92.6
CNN/LSTM	88.7	91.7

**Table 7:** Test accuracy results when training on the TrainU1 dataset with the CNN architecture while using only accelerometer measurements.

Database/Smartphone Location	HTA	TestU1
Pocket	94.1	92.3
Swing	91.3	91.8
Texting	92.0	93.9
Talking	93.8	92.1

**Table 8:** Test accuracy results when training on the TrainROU dataset while using both accelerometer and gyroscope measurements.

Architecture/Database	HTA	MSR	Test U1	Test RIDI	Test OXF
LSTM	90.3	92.0	99.5	99.4	99.2
CNN	96.7	94.8	99.3	99.4	99.1
GRU	90.1	90.1	99.4	98.2	98.8
CNN/LSTM	97.1	92.7	99.2	99.6	99.0

**Table 9:** Test accuracy results when training on the TrainROU dataset with the CNN architecture while using both accelerometer and gyroscope measurements.

Database/Smartphone Location	HTA	MSR	Test U1	Test RIDI	Test OXF
Pocket	96.2	94.8	98.7	99.3	99.1
Swing	96.3	N/A	99.3	N/A	N/A
Texting	98.4	N/A	99.7	99.4	99.1
Talking	97.1	N/A	99.2	N/A	N/A

**Table 10:** Test accuracy results when training on the TrainROU dataset while using only accelerometer measurements.

Architecture/Database	HTA	MSR	WIS	OWB	PAR	Test ROU
LSTM	90.6	82.0	79.6	93.8	95.1	97.5
CNN	94.7	72.4	82.8	94.4	99.2	96.9
GRU	98.5	61.1	78.4	93.3	97.4	95.4
CNN/LSTM	90.1	71.3	72.4	91.1	99.2	97.3

**Table 11:** Hyperparameter test accuracy results when training on the TrainROU dataset while using accelerometer measurements only.

Architecture/Database	HTA	MSR	WIS	OWB	PAR	Test ROU
CNN	92.4	84.5	84.9	93.1	96.6	97.2
CNN/LSTM	92.4	96.6	92.9	93.4	99.6	98.4

**Table 12:** Test accuracy results after hyperparameter tuning when training on the TrainROU dataset with the CNN/LSTM architecture while using accelerometer measurements only.

Database/Smartphone Location	HTA	MSR	WIS	OWB	PAR	Test ROU
Pocket	91.5	96.6	92.9	95.1	N/A	98.4
Swing	92.1	N/A	N/A	91.6	99.6	99.1
Texting	97.0	N/A	N/A	N/A	N/A	98.4
Talking	90.3	N/A	N/A	N/A	N/A	99.2

## Appendix A

In this appendix, the sample distribution of the four smartphone mode locations of each dataset is presented. Since the MSR and WIS datasets contain only pocket samples and the PAR dataset contains only swing samples, no figure is presented for them.
